# Female-specific SNP markers provide insights into a WZ/ZZ sex determination system for mud crabs *Scylla paramamosain*, *S. tranquebarica* and *S. serrata* with a rapid method for genetic sex identification

**DOI:** 10.1186/s12864-018-5380-8

**Published:** 2018-12-29

**Authors:** Xi Shi, Khor Waiho, Xincang Li, Mhd Ikhwanuddin, Guidong Miao, Fan Lin, Yueling Zhang, Shengkang Li, Huaiping Zheng, Wenhua Liu, Jude Juventus Aweya, Ghazali Azmie, Juliana C. Baylon, Emilia T. Quinitio, Hongyu Ma

**Affiliations:** 1 Guangdong Provincial Key Laboratory of Marine Biotechnology, Shantou University, 243 Daxue Road, Shantou, 515063 China; 2 STU-UMT Joint Shellfish Research Laboratory, Shantou University, Shantou, 515063 China; 3 Laboratory for Marine Fisheries Science and Food Production Processes, Qingdao National Laboratory for Marine Science and Technology, Qingdao, 266071 China; 4 East China Sea Fisheries Research Institute, Chinese Academy of Fishery Sciences, Shanghai, 200090 China; 5 Institute of Tropical Aquaculture, Universiti Malaysia Terengganu, 21030 Kuala Terengganu, Malaysia; 6 Division of Biological Sciences, College of Arts and Sciences, University of the Philippines, Visayas, 5023 Miagao, Philippines; 7 Aquaculture Department, Southeast Asian Fisheries Development Center, 5021 Tigbauan, Philippines

**Keywords:** *Scylla* spp., RAD-seq, Female-specific SNP markers, WZ/ZZ sex determination system, Genetic sex identification

## Abstract

**Background:**

Mud crabs, *Scylla* spp., are commercially important large-size marine crustaceans in the Indo-West Pacific region. As females have the higher growth rate and economic value, the production of all female stocks is extremely essential in aquaculture. However, the sex determination mechanism is still unclear. Development of sex-specific genetic markers based on next-generation sequencing proved to be an effective tool for discovering sex determination system in various animals.

**Results:**

Restriction-site associated DNA sequencing (RAD-seq) was employed to isolate sex-specific SNP markers for *S. paramamosain*. A total of 335.6 million raw reads were obtained from 20 individuals, of which 204.7 million were from 10 females and 130.9 million from 10 males. After sequence assembly and female-male comparison, 20 SNP markers were identified to be sex-specific. Furthermore, ten SNPs in a short sequence (285 bp) were confirmed heterozygous in females and homozygous in males in a large population by PCR amplification and sequencing. Subsequently, a female-specific primer was successfully designed according to the female-specific nucleotide which could amplify an expected band from females but not from males. Thus, a rapid and effective method for molecular sexing in *S. paramamosain* was developed, meanwhile, this method could successfully identify the sex of *S. tranquebarica* and *S. serrata*. Finally, nine and four female-specific SNP markers were detected in *S. tranquebarica* and *S. serrata*, respectively.

**Conclusions:**

Sex-specific SNP markers were firstly identified in crab species and showed female heterogamety and male homogamety, which provided strong genetic evidence for a WZ/ZZ sex determination system in mud crabs *S. paramamosain*, *S. tranquebarica* and *S. serrata*. These findings will lay a solid foundation for the study of sex determination mechanism, sex chromosome evolution, and the development of mono-sex population in crustaceans.

**Electronic supplementary material:**

The online version of this article (10.1186/s12864-018-5380-8) contains supplementary material, which is available to authorized users.

## Background

Sex determination mechanisms of animals are remarkably diverse and complicated, and they are attracting considerable interest due to its great implication in both theory and practice [1–3]. Unlike vertebrates, the sex determination mechanisms of crustaceans are more varied and frequently affected by genetic and/or environmental factors [4, 5]. Among crustaceans, several crab species, including *Plagusia dentipes, Eriocheir japonicus* and *Hemigrapsus sanguineus*, are thought to have a XY/XX sex determination system based on karyotype studies [6–8]. However, a recent study suggested that Chinese mitten crab (*Eriocheir sinensis*) exhibited a WZ/ZZ sex determination system according to quantitative trait locus location of the gender phenotype [5]. Meanwhile, the WZ/ZZ sex determination system has also been observed in other crustaceans, such as kuruma prawn (*Penaeus japonicus*) [9], Pacific white shrimp (*Litopenaeus vannamei*) [10], and giant freshwater prawn (*Macrobrachium rosenbergii*) [11]. Importantly, studies on the genetic basis of sex determination mechanism are the foundation towards future sex manipulation biotechnologies, including the development of mono-sex population [2, 12], especially for those crabs with significant sexual dimorphism, such as caribbean king crab (*Mithrax spinosissimus*) [13] and Japanese mitten crab (*Eriocheir japonica*) [14]. So far, the sex determination mechanism remains unclear in most aquaculture crustacean species, which have therefore obviously limited its potential application in the aquaculture sector.

Generally, there are three popular types of techniques for studying sex determination mechanism, i.e. cytogenetic approaches, breeding experiments, and sex-specific molecular markers [15]. The application of cytogenetic analysis is limited because some species lack visually heteromorphic sex chromosomes, while breeding experiments are mainly focused on several common species, and therefore impracticable for many species. The use of sex-linked or sex-specific markers is regarded as a powerful tool for well-understanding sex determination system in most species [2, 12, 16].

Over the last few decades, various genetic approaches have been successfully applied to identify sex-specific DNA sequences or markers in a range of aquaculture fish and crustacean species. For example, random amplified polymorphic DNA (RAPD) for turbot (*Scophthalmus maximus*) [17], amplified fragment length polymorphism (AFLP) for swimming crab (*Portunus trituberculatus*) [18] and Pacific bluefin tuna (*Thunnus orientalis*) [19], as well as simple sequence repeat (SSR) markers for half-smooth tongue sole (*Cynoglossus semilaevis*) [20, 21] and rock bream (*Oplegnathus fasciatus*) [22]. Currently, with the rapid development of next-generation sequencing (NGS) technologies, some novel methods have been developed for exploring sex-associated DNA markers [3, 23]. NGS-based marker systems allow highly efficient DNA markers development within a short period [24]. Restriction-site associated DNA sequencing (RAD-seq) is based on NGS technologies and can discover massive single nucleotide polymorphisms (SNPs) in various species by sequencing parts of the genome at high depth, without a reference genome [23, 25–27]. Recently, RAD-seq was successfully used to develop sex-specific markers in aquaculture species, such as Atlantic halibut (*Hippoglossus hippoglossus*) [28], European sea bass (*Dicentrarchus labrax*) [25], and silver carp (*Hypophthalmichthys molitrix*) [3].

Mud crabs (*Scylla* spp.) are highly valuable commercial aquaculture species and fishery resources in the Indo-West Pacific region, such as China [29], Thailand [30], Vietnam [31], Philippines [32] and Malaysia [33]. The aquaculture output of mud crab in China reached approximately 148,977 tons, the top among all marine commercial crabs [34]. Although the farming and fishing output of mud crab increased tremendously in recent years, the current scale of production is still too inadequate to meet the market demand [35]. A major challenge in mud crab aquaculture industry presently is how to develop a set of fast and viable seed production techniques so as to improve supply [36]. Additionally, *Scylla paramamosain*, one of mud crab species, exhibits significant sexual dimorphism in growth rate and body size, with females growing faster and having higher nutritive value than males [37]. Thus, it is essential to develop sex-linked markers for production of mono-sex breeding, shortening farming duration, as well as understanding the genetic basis of sex determination system [3, 28]. However, there is currently no available genetic information on sex determination system for these economically important crustacean species*.*

In the present study, RAD-seq technology was employed to identify and characterize sex-specific SNP markers in mud crab *S. paramamosain*. A rapid and reliable molecular method based on the female-specific SNP markers was developed to identify the genetic sex of individuals at early developmental stages. Further, the developed PCR-based genetic sex identification method was applied in two other mud crab species, *S. tranquebarica* and *S. serrata*. The results suggest a WZ/ZZ sex determination system in *S. paramamosain*, *S. tranquebarica* and *S. serrata*. These findings will provide new insights into the mechanism of sex determination in brachyuran crabs, as well as facilitate the development of mono-sex population of mud crab and related crustacean species.

## Results

### RAD sequencing

A total of 335,600,178 raw reads were generated after RAD-seq, with 204,685,780 for females and 130,914,398 for males (Table 1). After filtering low quality reads, 172,889,526 clean reads for females and 111,335,216 clean reads for males were obtained, with an average of 14,211,237 per sample. The average read length of each sample ranged from 126.2 to 134.1 bp, while the average clean reads rate of all samples was 83.75%. Based on the read quality and quantity, female sample named “F2A” was selected as a reference, and its clean reads were clustered and assembled (Additional file 1). After filtering, 272,347 tags were used for assembly, retrieving 69,799,531 bp data and 242,137 contigs. The contig N50 was 300 bp and GC content was 40.50% (Additional file 1).

**Table 1:** Summary of RAD-seq data for ten male and ten female *Scylla paramamosain*

Sample	Barcode	Raw reads	Raw bases	Clean reads	Clean bases	Average read length (bp)	Clean reads rate (%)	Clean bases rate (%)
Female								
F1A	ACGTA	24,557,238	3,646,738,259	21,847,092	2,757,935,713	126.2	88.96%	75.63%
F2A	ACTGC	29,997,130	4,454,521,671	26,462,942	3,348,677,805	126.5	88.22%	75.17%
F9A	AGAGT	18,726,918	2,780,858,197	16,501,732	2,088,286,211	126.5	88.12%	75.10%
F1C	ACGTA	23,229,362	3,449,530,993	19,460,818	2,596,836,132	133.4	83.78%	75.28%
F2C	ACTGC	9,141,424	1,357,421,834	6,843,196	907,264,996	132.6	74.86%	66.84%
F3C	AGAGT	31,790,388	4,720,690,658	26,567,472	3,549,305,187	133.6	83.57%	75.19%
F5C	ACCAT	34,198,658	5,078,387,213	28,275,808	3,771,444,128	133.4	82.68%	74.26%
F6C	ACGTA	4,878,130	724,383,091	3,886,802	518,537,398	133.4	79.68%	71.58%
F8C	ACTGC	19,956,292	2,963,456,904	16,356,092	2,192,594,425	134.1	81.96%	73.99%
F9C	AGAGT	8,210,240	1,219,076,028	6,687,572	896,711,413	134.1	81.45%	73.56%
Subtotal	–	204,685,780	30,395,064,848	172,889,526	22,627,593,408	–	–	–
Subaverage	–	20,468,578	3,039,506,485	17,288,953	2,262,759,341	131.4	83.33%	73.66%
Male								
M1A	ACCAT	13,510,792	2,006,303,898	11,718,538	1,480,652,251	126.4	86.73%	73.80%
M3A	ACGTA	4,760,476	706,930,686	3,924,978	515,736,898	131.4	82.45%	72.95%
M5A	ACTGC	9,174,030	1,362,343,455	7,514,458	990,015,495	131.7	81.91%	72.67%
M6A	AGAGT	11,568,106	1,717,863,741	9,898,912	1,304,979,357	131.8	85.57%	75.97%
M8A	ACCAT	12,354,600	1,834,658,100	10,442,804	1,378,972,481	132.1	84.53%	75.16%
M2C	ACCAT	10,770,088	1,599,284,962	8,790,688	1,178,460,368	134.1	81.62%	73.69%
M3C	ACGTA	30,743,266	4,565,350,471	27,115,768	3,471,064,019	128.0	88.20%	76.03%
M4C	ACTGC	19,284,772	2,863,734,056	16,257,120	2,114,310,426	130.1	84.30%	73.83%
M6C	AGAGT	6,010,814	892,543,605	4,944,440	640,757,057	129.6	82.26%	71.79%
M7C	ACCAT	12,737,454	1,891,463,037	10,727,510	1,395,530,197	130.1	84.22%	73.78%
Subtotal	–	130,914,398	19,440,476,011	111,335,216	14,470,478,549	–	–	–
Subaverage	–	13,091,440	1,944,047,601	11,133,522	1,447,047,855	130.5	84.18%	73.97%
Total	–	335,600,178	49,835,540,859	284,224,742	37,098,071,957	–	–	–
Average	–	16,780,009	2,491,777,043	14,211,237	1,854,903,598	131.0	83.75%	73.81%

### Identification of sex-specific SNP markers

**Fig. 1:**
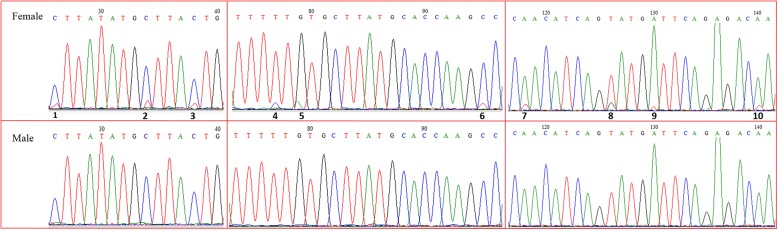
Ten female-specific SNP markers in the sequencing chromatograms of the sex-related sequence of *Scylla paramamosain*. SNP1: C/T; SNP2: C/T; SNP3: C/T; SNP4: T/C; SNP5: G/A; SNP6: C/T; SNP7: A/T; SNP8: T/G; SNP9: A/T; SNP10: A/T

The reads of 20 individuals were mapped to the reference sample “F2A” (Additional file 2), with the mapping rate for each individual varying from 49.73 to 80.34%. After alignment analysis, 1,780,706 polymorphic SNP markers across the 20 individuals were obtained. These SNP markers were subjected to sex-specific marker identification, and as a result, a total of 20 sex-specific SNP markers (Table 3) were identified in 11 contigs by comparing the SNPs between sexes. Next, 10 primer pairs were designed to amplify the corresponding sequences (Table 2). After testing, primer 11,508 (Table 2) could successfully amplify Cluster_384014 with 285 bp in length (Table 3), which was regarded as a sex-related sequence. PCR amplification and sequencing analysis showed that this sequence was completely identical between sexes except 10 SNPs. These 10 SNPs were heterozygous (including C/T, G/A, A/T, T/G) in all females, but homozygous in all males (Fig. 1), indicating that the sex determination system in *S. paramamosain* is WZ/ZZ with females being heterogametic. These SNP markers were then successfully validated by PCR assay and sequencing using additional 195 specimens (106 females and 89 males).

**Table 2:** Primers used for the validation of female-specific SNP markers, the extension of female-related sequence and the PCR-based genetic sex identification method

Name	Reference sequence	Primers (5′-3′)	Annealing temperature (°C)
4307 ^a^	Cluster_121142	F: TTTGCTTTTTTTGTCTTATGGTTC	52
		R: AAACAAATTTACTGAAAACGTGTCT	
8966 ^a^	Cluster_136335	F: TATAGAGTGCTTTGCATCAATT	51
		R: TTCAAAACAAAATTACTGAAAAC	
6579 ^a^	Cluster_164559	F: CCTTGTGGTTCTTTTGAAC	50
		R: AACGAAATTACTGAAAACTTG	
9673 ^a^	Cluster_22418	F: TCATAGGTACCAAGATGCC	55
		R: GGTTTCTCGTAATGTCGTT	
8171 ^a^	Cluster_31265	F: GAAAACGTGTCTTCCCAGTG	54
		R: ATATACACGAAGGTTTGCGTT	
11,508 ^a^	Cluster_384014	F: GCTTATCATAGTTATTGCCTTGT	53
		R: TGCACTCATGCTGGATTTT	
1888 ^a^	Cluster_39896	F: GATTTCATCATCACCGACG	57
		R: GCAATTCTTGTCTGAGCATG	
4225 ^a^	Cluster_4545	F: CAGCCCCGACATTAAGGC	56
		R: ATATACTGCAATTCTAATGCCAGG	
9132 ^a^	Cluster_4856	F: ATTATTCTGGTGACTAACA	55
		R: GCTAAAACTTCTTTATAGAG	
5476 ^a^	Cluster_71436	F: CTATATTGTTAATTGTTTTGGTGAC	53
		R: TCATCTTCATAGGTACCAATATCA	
FLFE-1 ^b^	Genome sequence	F: GTACTCTTTAATCAGTTTGTGCCCAT	53
		R: CTGCCAGTGATTCAGTGACTTAGC	
FLFE-2 ^b^	Genome sequence	F: ATGTTTATTGTGTTGTTCAGTGTTGTCT	53
		R: CGAGGGTTACTGTAGTTAATGGC	
SPC ^c^	Extended sequence of female-specific fragment	F: GTTCTGCTTATCATAGTTATTGCCTTG	65
		R: CTGCCAGTGATTCAGTGACTTAGC	
SPFS ^c^	Cluster_384014	F: **C**TTAGTATATCACAAC**T**ACATCAG**G**ATG**T**	65
		R: AAGATGCTTGCTGTCTCATTGGT	

^a^Primers used for the development and validation of female-related SNP markers; ^b^ Primers used for the extension of female-related sequence, FLFE: female-related fragment extension; ^c^ Primers used for PCR-based genetic sex identification method, SPC: *Scylla paramamosain* control primer, SPFS: *S. paramamosain* female-specific primer, letters in bold were four mismatch nucleotides

**Table 3:** Marker names, positions and sequences of 20 candidate sex-related SNP markers in *Scylla paramamosain*

No.	Marker Name	SNP Position			Reference Base	Alternative Base
		Sequence	Sequence length	Position		
1	SNP-4307	Cluster_121142	306	140	T	A
2	SNP-8966-1	Cluster_136335	294	242	T	C
3	SNP-8966-2	Cluster_136335	294	265	G	A
4	SNP-6579-1	Cluster_164559	299	205	C	T
5	SNP-6579-2	Cluster_164559	299	236	T	C
6	SNP-9673-1	Cluster_22418	246	128	C	T
7	SNP-9673-2	Cluster_22418	246	133	A	T
8	SNP-9673-3	Cluster_22418	246	231	T	A
9	SNP-8171	Cluster_31265	290	214	C	G
10	SNP-11508	Cluster_384014	285	70	T	C
11	SNP-1888	Cluster_39896	319	294	T	C
12	SNP-572	Cluster_40,991	297	285	T	A
13	SNP-4225-1	Cluster_4545	317	98	A	G
14	SNP-4225-2	Cluster_4545	317	143	T	C
15	SNP-4225-3	Cluster_4545	317	285	T	A
16	SNP-9132-1	Cluster_4856	320	198	C	T
17	SNP-9132-2	Cluster_4856	320	226	T	C
18	SNP-5476-1	Cluster_71436	243	13	T	C
19	SNP-5476-2	Cluster_71436	243	133	G	C
20	SNP-5476-3	Cluster_71436	243	148	T	A

### Development of the PCR-based genetic sex identification method

**Fig. 2:**
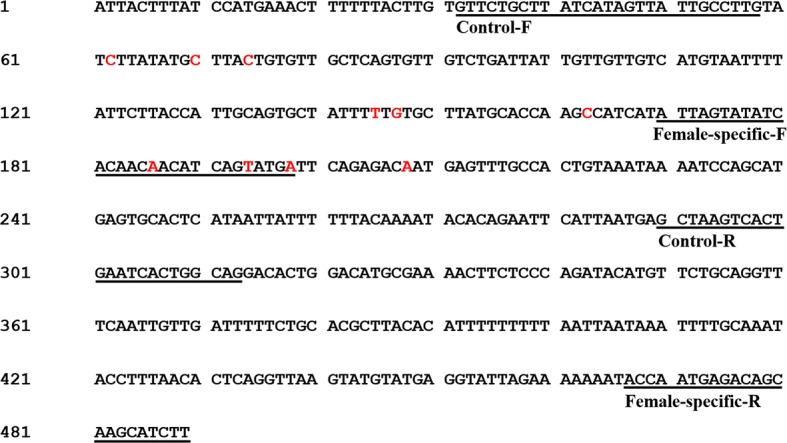
Part of female-related DNA sequence cloned based on the genome sequence of *Scylla paramamosain.* The red color letters in the sequence indicate the ten female-specific SNPs, where SNP1: C/T; SNP2: C/T; SNP3: C/T; SNP4: T/C; SNP5: G/A; SNP6: C/T; SNP7: A/T; SNP8: T/G; SNP9: A/T; SNP10: A/T. The underlined text indicated the primer binding sites in the PCR-based genetic sex identification method, where Control-F and Control-R were the control primer (SPC) binding sites, and Female-specific-F and Female-specific-R were the female-specific primer (SPFS) binding sites

**Fig. 3:**
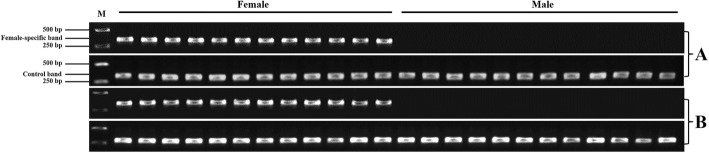
Application of the PCR-based genetic sex identification method in *Scylla paramamosain*. Female-specific band (320 bp): PCR products amplified with the female-specific primer (SPFS); Control band (282 bp): PCR products amplified with control primer (SPC); M: marker; **a**: the agarose gel electrophoresis results for 12 females and 12 males cultured in a pond at Shantou, China. **b**: the agarose gel electrophoresis results for another 12 females and 12 males cultured in a pond at Shantou, China

First, the sequence (Cluster_384014) which contained the 10 female-specific SNP markers was lengthened to 2315 bp by comparing with the whole genome sequences of *S. paramamosain* (unpublished data) and molecular cloning. This sequence was submitted to GenBank database under the accession number of MH133208. Subsequently, the PCR-based genetic sex identification method was successfully developed based on the lengthened sequence and the female-specific SNP markers. A pair of female-specific primers (SPFS, Table 2, Fig. 2) produced a stable female-specific band with 320 bp in length in females, but not in males. Meanwhile, a pair of control primers (SPC, Table 2, Fig. 2) produced a 282 bp long band in both females and males. Four nucleotides mismatches were artificially created in the female-specific primer, of which three were at the female-specific SNPs, SNP 7, SNP 8 and SNP 9, and one was at the 5′ end of the primer (Table 2, Fig. 2). A total of 96 specimens (48 females and 48 males) were used to determine the accuracy and precision of the PCR-based genetic sex identification method. The results showed a 100% accuracy for identification of genetic sex of *S. paramamosain*. (Fig. 3 and Additional file 3).

### Application of the molecular genetic sex identification method to individuals at early developmental stages

The PCR-based genetic sex identification method was applied to determine the sex and sex ratio of 180 offspring (unknown sex) of *S. paramamosain* from a full-sib family at three different early developmental stages, i.e. megalopa stage (M), the first crablet stage (C1), and the second crablet stage (C2). At these three developmental stages, the number of females were 30, 24, 25, and the number of males were 30, 36, 35, respectively (Table 4). The female/male ratio at each developmental stage was 1.00, 0.67 and 0.71, respectively, with the total sex ratio being 0.78 (Table 4). There was a tendency toward a lower female/male ratio with the growth and development of *S. paramamosain.* Chi-square tests showed that the sex ratio at each developmental stage and the total sex ratio did not exhibit significant differences with the 1:1 separation ratio (*P* > 0.05) (Table 4).

**Table 4:** The sex ratio of *Scylla paramamosain* at three different early developmental stages

Developmental stage	Number of offspring	Number of females	Number of males	Sex ratio ^a^	*P* ^b^
Megalopa stage (M)	60	30	30	1.00	1.000
First crablet stage (C1)	60	24	36	0.67	0.271
Second crablet stage (C2)	60	25	35	0.71	0.360
Total	180	79	101	0.78	0.245

^a^Sex ratio: female: male. ^b^ *P* value with Chi-square tests

### Cross-species application of the molecular genetic sex identification method and identification of sex-specific SNP markers

**Fig. 4:**
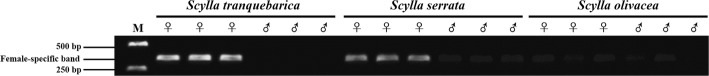
Application of the PCR-based genetic sex identification method in three other species of genus *Scylla*. Female-specific band (320 bp): PCR products amplified with female-specific primer (SPFS); The PCR-based genetic sex identification method was effective for *S. tranquebarica* and *S. serrata,* but not for *S. olivacea*, even though an extremely faint band exhibited in male individuals of *S. serrata*

To test the feasibility of the newly developed molecular sexing method in three other species of genus *Scylla,* the female-specific primer (SPFS, Table 2) was used to amplify the potential bands in *S. tranquebarica, S. serrata* and *S. olivacea*. Interestingly, the results showed that this method worked well in *S. tranquebarica* and *S. serrata* despite an extremely weak band exhibited in the males of *S. serrata* (Fig. 4). To investigate whether the sex-specific SNP markers exist in these three species, primer 11,508 (Table 2) was employed to amplify the corresponding fragment and then sent for sequencing. After sequence comparison, nine (SNP2-SNP10, Table 5) and four (SNP2, SNP3, SNP9, SNP10, Table 5) sex-specific SNP markers were identified from *S. tranquebarica* and *S. serrata*, respecitively. The genotypes of these sex-specific markers were identical to those detected in *S. paramamosain*, being heterozygous in all females, but homozygous in all males. In addition, no sex-specific SNP markers were observed in *S. olivacea* (Table 5)*.*

**Table 5:** The nucleotide polymorphisms at ten SNP markers of genus *Scylla*

Species	Gender	Nucleobases in each SNP marker									
		SNP1	SNP2	SNP3	SNP4	SNP5	SNP6	SNP7	SNP8	SNP9	SNP10
*Scylla paramamosain*	F	C/T	C/T	C/T	T/C	G/A	C/T	A/T	T/G	A/T	A/T
	M	C	C	C	T	G	C	A	T	A	A
*Scylla tranquebarica*	F1	C	C/T	C/T	T/C	G/A	C/T	A/T	T/G	A/T	A/T
	F2	C	C/T	C/T	T/C	G/A	C/T	A/T	T/G	A/T	A/T
	F3	C	C/T	C/T	T/C	G/A	C/T	A/T	T/G	A/T	A/T
	M1	C	C	C	T	G	C	A	T	A	A
	M2	C	C	C	T	G	C	A	T	A	A
	M3	C	C	C	T	G	C	A	T	A	A
*Scylla serrata*	F1	C	C/T	C/T	T	G	C	A/C	T	A/T	A/T
	F2	C	C/T	C/T	T	G	C	A/C	T	A/T	A/T
	F3	C	C/T	C/T	T	G/A	C	A	T/G	A/T	A/T
	M1	C	C	C	T	G	C	A	T	A	A
	M2	C	C	C	T	G	C	A	T	A	A
	M3	C	C	C	T	G	C	A	T	A	A
*Syclla olivacea*	F1	C	C	C	T	G	C	A/T	T	A	A/T
	F2	C	C	C	T	G	C	A	T	A	A/T
	F3	C	C/T	C	T/C	G/A	C/T	A/T	T/G	A	A/T
	M1	C	C	C	T	G	C	A	T	A	A/T
	M2	C	C	C	T	G	C	A	T/G	A	A/T
	M3	C	C	C	T	G/A	C	A/T	T/G	A	A/T

*F* female, *M* male

## Discussion

Studies on sex determination system of crabs are much limited. In the latter part of the last century, the XY/XX sex determination system was suggested for crabs [8, 38], but recently, the WZ/ZZ sex determination system was found in Chinese mitten crab [5]. To the best of our knowledge, this study is the first to focus on the sex determination system of genus *Scylla*. Here, 10 female-specific SNP markers were verified from DNA sequence in 195 individuals of *S. paramamosain*. These identified SNP markers were heterozygous in all females but homozygous in all males, suggesting a WZ/ZZ female heterogametic sex determination system in *S. paramamosain.* Additionally, the female-specific SNP markers were detected in a short DNA sequence of both females and males, which indicated the W and Z chromosomes of *S. paramamosain* are not fully differentiated from each other at DNA level and there are still many homologous loci, as suggested by Cui et al. [5]. The stable sex-specific genetic markers deteced in this study may due to the meiotic recombination suppression between sex chromosomes [39–41].

Sex determination system is essential not only for the study of reproductive biology and the process of genome evolution [5, 42], but also for the development of sex manipulation techniques, especially in economically important species with obvious sexual dimorphic characters such as mud crab. Given the large number of chromosomes in crustaceans, to elucidate the sex determination system using cytogenetic investigations and high-resolution linkage map is challenging [5]. Fortunately, sex-linked DNA markers provides an alternative tool to study sex determination system and it has shown great potential application in various aquaculture species [3, 27, 43]. In this study, the sex-linked SNPs provide a strong evidence for chromosomal sex determination system and serve as a crucial foundation for development of mono-sex population of *S. paramamosain* [2, 12]. Additionally, these sex-linked markers will be helpful for identifying sex-related contigs from whole genome assemblies, thereby broadening our current knowledge of sex chromosome genes and evolution [15].

The ability to correctly identify sex of animals is of critical importance in ecological and evolutionary studies, as well as for artificial breeding and farming purposes [44, 45]. Several approaches have been developed for sex identification in animals, such as behavior observation [46], morphological and external characteristics [47], ultrasonography [48] and hormone levels [49]. However, these methods are prone to high error rates and sometimes technically complex [45]. Genetic sex identification is an essential sex determination tool when the sex of an organism can not be discriminated morphologically [12]. In the last decade, sex-specific DNA markers have been successfully used to distinguish sex of different species, especially in animals at their early developmental stages without obvious sexual traits [12, 21, 43, 50]. The PCR-based method for genetic sex identification is believed to be expedient, time-saving and cost-effective [12, 45, 51].

The sex of *S. paramamosain* is indistinguishable by morphological traits at their early developmental stages, especially younger than one-month old. The lack of sex identification methods have tremendously limited the study on sex determination and differentiation between males and females. Thus, the molecular sex identification method for *S. paramamosain* is essential for these reasons. Among the PCR-based sex identification methods, sex-specific primers were usually designed with different strategies, for example, sex-specific DNA sequence [3, 50], two mismatched nucleotides in the forward primer [12], and DNA sequence deletion between sexes [43]. In the present study, four nucleotide mismatches containing three female-specific SNP markers were artificially created that gave positive PCR amplification in females but not in males. Importantly, the mismatch in the 3’ end of the female-specific primer played a vital role in this method because the initial binding site with DNA template is at the 3’ end of the primer. Thus, in addition to providing a novel method for determining the sex of *S. paramamosain*, especially during larval and juvenile stages, this method also provided new insights into the application of sex-specific SNP markers to genetic sex identification of other brachyuran species in the future, and it should be beneficial for the marker-assisted sex control breeding and aquaculture [21, 50, 52].

Further, the investigation of sex and sex ratio of individuals at three early developmental stages (M, C1 and C2) from a full-sib family showed no significant deviation from the expected ratio of 1:1, which was consistent with the genetic sex determination mechanism suggested in this study. However, the number of females decreased with the growth and development of *S. paramamosain*. In our previous study, the number of female *S. paramamosain* at two-month old (older than C2) was also lower than males, but the sex ratio significantly skewed towards females from three-month old to four-month old (unpublished data). The variation of the sex ratio during the process of growth and development could be due to the known aggressive and cannibalistic behavior of *Scylla* spp., and the growing competition for food and spouses, especially for male crabs [33], which leads to injury and death of males.

Sex-specific markers can also provide insights into sex chromosome conservation and evolution [15, 27]. The same sex-specific markers and sex-determining DNA sequence were identified and verified in bighead carp, silver carp and grass carp by 2b-RAD sequencing, suggesting that the same pathways might be involved in sex determination systems [3]. In this study, *S. tranquebarica* and *S. serrata* were proved to have nine and four identical female-specific SNP markers with *S. paramamosain,* respectively, suggesting that these three crab species may have a homologous nascent W chromosome and share the most recent common ancestor [3]. Therefore, the WZ/ZZ sex determination system is suggested for *S. paramamosain*, *S. tranquebarica* and *S. serrata*. While the data obtained in the present study was unable to draw conclusion on the genetic sex determination system of *S. olivacea* because no sex-specific markers were observed. However, the sex-specific SNP markers may exist in other genome regions of *S. olivacea*, hence, further studies should be carried out in the future to explore this.

Although mud crabs have been captured and cultured for over 110 years, the taxonomy of genus *Scylla* was controversial until the revision by Keenan et al. in 1998 [53], based on genetic variations, external morphology and morphometric characters analysis [54]. Phylogenetic study on genus *Scylla* based on COI sequence of mtDNA showed that *S. paramamosain* is genetically closest to *S. tranquebarica,* and then to *S. serrata*, while, the largest genetic distance was found with *S. olivacea* [55, 56]. In the present study, the number of identical female-specific SNP markers were found to be nine between *S. paramamosain* and *S. tranquebarica*, four between *S. paramamosain* and *S. serrata*, but none between *S. paramamosain* and *S. olivacea*, an observation which is supported by the phylogenetic relationships of genus *Scylla* as determined by previous studies [55, 56]. Nevertheless, we suggest that for deeply understanding the evolution and phylogenetic relationships of genus *Scylla*, more research should be carried out.

## Conclusions

RAD-seq technology has proven to be a good tool for the identification of sex-specific markers in non-model organisms. To the best of our knowledge, this is the first study to identify female-specific SNP markers in mud crab (*S. paramamosain*) that showed heterozygous in females but homozygous in males. Subsequently, a rapid and effective method for molecular genetic sex identification was developed, by which, the sex of *S. paramamosain*, *S. tranquebarica* and *S. serrata* could be distinguished with a 100% accuracy. Moreover, nine and four identical female-specific SNP markers similar to those in *S. paramamosain* were identified in *S. tranquebarica* and *S. serrata*, respectively. These findings provided a solid evidence for a WZ/ZZ sex determination system in these three mud crab species. This study will not only further our understanding of sex determination mechanism and genome evolution of genus *Scylla*, but also facilitate mono-sex breeding and aquaculture of these crab species.

## Methods

### Samples collection and DNA extraction

Twenty wild *S. paramamosain* (10 males and 10 females), randomly collected from the inshore of Hainan Province, China, were used to determine the potential sex-specific SNP markers by RAD-seq. Another 195 specimens (106 females and 89 males) were collected and used to confirm the sex-specific SNP markers, of which 115 were from the inshore of Shantou and 80 were from a culture pond in Hainan. In addition, 96 specimens (48 females and 48 males) were collected from two culture ponds in Shantou and Raoping for verification of the molecular sex identification method developed based on the sex-specific SNP markers. Another 180 young progenies with unknown sex from a full-sib family were sampled and used to distinguish the sex and sex ratio using the developed molecular sex identification method. Among these progenies, 60 individuals were at megalopa stage (M), 60 individuals were at the first crablet stage (C1), and 60 individuals were at the second crablet stage (C2). In order to test the feasibility of the sex-specific SNP markers and the developed sex identification method in other *Scylla* species, *S. tranquebarica* (*N* = 20, 10 males and 10 females) and *S. olivacea* (*N* = 6, 3 males and 3 females) were collected from Setiu Wetlands, Terengganu, Malaysia, whereas *S. serrata* (*N* = 6, 3 males and 3 females) was caught from Iloilo, Philippines. The sex of these crabs was identified based on the shape of the abdomen. Females have wider and more globular abdomens, whereas males have narrow and straight abdomens [57].

Before sampling, the crabs were placed on ice for anesthetization (about 10 min). Muscle tissues were frozen in liquid nitrogen and preserved at − 80 °C until DNA extraction. The whole body of the individuals at early developmental stages (M, C1 and C2) were used for DNA extraction due to their small size. Genomic DNA was extracted using TIANamp Marine Animals DNA kit (Tiangen Biotech Co. Ltd., Beijing, China) and treated with RNase A to remove residual RNA. The DNA quality was assessed by agarose gel electrophoresis and quantified using a Qubit fluorometer (Life Technologies).

### RAD library preparation and sequencing

The RAD library was prepared following previous methods [58–60] with slight modifications. Briefly, approximately 1 μg of genomic DNA from each individual was digested with *Eco*RI-HF. The P1 adapter which contains individual-specific index sequence of 5 bp long barcode (Table 1) was ligated to the purified products of restriction reaction. The ligated samples were then pooled, purified and randomly sheared to short fragments with an average size of 350 bp using Bioruptor (Diagenode, Liège, Belgium). The P2 adapter which contains a 3′ dT overhang was ligated to the sheared DNA fragments. Furthermore, the ligation mix with 300 to 500 bp was purified using the MinElute Gel Extraction Kit (Qiagen). The library was further enriched by high-fidelity PCR using P1 and P2 adapter primers. Finally, the PCR products comprising between 300 and 500 bp were purified and sequenced (2 × 150-bp paired-end reads) on Illumina Hiseq 3000 platform. Raw data generated in this study has been submitted to the NCBI Short Read Archive (SRA) under the accession number SRP135178.

### Sequence analysis and sex-specific SNPs discovery

The raw reads from the Illumina Hiseq were first sorted according to the individual-specific index sequence, and then the indexes and low-quality reads were removed by Trim Galore software (http://www.bioinformatics.babraham.ac.uk/projects/trim_galore/). Quality control analysis was performed to assess the length distribution and GC content of the clean reads using FastQC software (http://www.bioinformatics.babraham.ac.uk/projects/fastqc/). Individual which named “F2A” has the best sequencing quality and quantity, so clean reads from it were clustered for reference using the software CD-HIT-EST (http://weizhongli-lab.org/cd-hit/) under the following thresholds: sequence identity ≥97%, minimum coverage 10, and maximum coverage 400, with the cluster analysis results shown in Additional file 1. After the cluster analysis, clean reads were assembled to contigs using the Spades software (http://cab.spbu.ru/software/spades/) with k-mer size of 21, 33 and 55. The contigs shorter than 150 bp were excluded from assembly. The assembled sequences of the sample “F2A” were regarded as a reference, with reads from each sample aligned to the assembled reference of “F2A” using Burrows-Wheeler Alignment Tool (BWA) [61]. The alignment results were processed using SAMtools [62] to identify candidate genetic variants among all sequenced samples, including both SNPs and INDELs. Bcftools and Vcfutils.pl were used to filter variants with the default parameters, such as the minimum root-mean-square read mapping quality of 10, a minimum depth of 2 and the minimum number of read supporting the variant of 2. The SNPs of males and females were summarized respectively, and then the sex-specific SNP markers were identified based on the SNP comparison between males and females. Only SNP markers with a polymorphic genotype associated with the sex were regarded as sex-specific markers.

### Development and verification of sex-specific SNP markers

Based on the results of RAD-seq, 11 contigs were found to contain sex-specific SNPs. A total of ten pairs of primers (Table 2) were designed by the Primer Premier 5.0 software, with the exclusion of one contig (cluster 40,991, Table 3) due to the near-end position of the sex-specific SNP marker. PCR amplification was then used to validate the specificity of each sex-specific SNP marker. The PCR reactions were carried out with a total volume of 25 μl, containing 50 ng of template DNA, 2.5 μl of 10 × PCR buffer, 2.0 μl of dNTPs (2.5 mM each), 0.5 μl of each primer (10 μM), 0.5 μl of *Taq* DNA polymerase (5 U/μl, TransGen Biotech, Beijing, China) and water to the final volume. PCR programs were as follows: an initial denaturation step at 94 °C for 5 min; then 34 cycles of 94 °C for 30 s, annealing temperature of primer pair for 30 s, and 72 °C for 40 s; and a final extension step at 72 °C for 7 min. The amplified products were separated by 1% agarose gel electrophoresis, and the expected PCR fragments were directly sequenced in both directions by Shanghai Sangon Biotechnology Co., Ltd. (Shanghai, China). The sex-specific SNP markers were determined manually by the peaks and their colors in the chromatogram. Among the sequences, one containing sex-specific SNPs with the best quality and quantity was regarded as sex-related sequence, and was used for further analysis. Furthermore, the sex-related sequence was amplified and the sex-specific SNP markers were verified using 195 other specimens (106 females and 89 males) with known sex. The PCR products were sequenced and the sex-specific SNPs were genotyped as described above.

### Development of a rapid method for genetic sex identification

First, the sex-related sequence was aligned to the genome of *S. paramamosain* (unpublished data), obtaining a longer sequence. The obtained sequence was then verified through molecular cloning and sequencing. The primers (FLFE-1 and FLFE-2, Table 2) used for DNA cloning were designed based on the genome sequences. Next, in order to develop a rapid PCR-based method to identify genetic sex of *S. paramamosain*, a pair of female-specific primers (SPFS, Table 2) was designed based on the sex-specific SNP markers, which is expected to amplify the sex-specific SNP regions only in females and show female-specific band on agarose gel electrophoresis. Meanwhile, a pair of control primers (SPC, Table 2) were designed based on the longer sex-related sequence and regarded as a control to avoid the presence of false-negative results, which can amplify the same size fragment in both males and females. The optimized PCR parameters of the PCR-based genetic sex identification method are as follows: total PCR reaction volume of 20 μl, containing 75 ng of template DNA, 2.5 μl of 10 × PCR buffer, 1.0 μl of dNTPs (2.5 mM each), 0.25 μl of each primer (10 μM), 0.4 μl of *Taq* DNA polymerase (5 U/μl, TransGen Biotech, Beijing, China) and water to the final volume. The PCR program was as follows: an initial denaturation step at 94 °C for 5 min; then 35 cycles of 94 °C for 30 s, 65 °C for 30 s, and 72 °C for 40 s; and a final extension step at 72 °C for 7 min. PCR amplification was conducted in 96 specimens (48 females and 48 males) to verify the PCR-based genetic sex identification method.

### Application of the PCR-based genetic sex identification method for determining the sex of individuals at early developmental stages

The sex of 180 individuals of *S. paramamosain* from a full-sib family at three different early developmental stages, i.e. M, C1 and C2, was identified by the PCR-based genetic sex identification method. The sex ratio at each developmental stage was estimated according to the identification result. Chi-square tests were used to estimate the fitness of sex ratio (female/male) to the expected 1:1.

### Cross-species application and verification of sex-specific SNP markers

The sex-specific SNP markers identified from *S. paramamosain* were cross-species tested in three other *Scylla* species, i.e. *S. tranquebarica*, *S. serrata* and *S. olivacea*, using PCR amplification and sequencing methods as described above. To test the feasibility of the newly developed molecular sex identification method, PCR amplifications were also carried out (Table 2) in these three other *Scylla* species.

### Data analysis

Chi-square tests were performed using the SPSS 21.0 for Windows (SPSS, Michigan Avenue, Chicago, IL, USA), and differences were considered statistically significant at *P* < 0.05.

## Additional files

Additional file 1: The cluster analysis and RAD assembly of sample “F2A”. (DOCX 14 kb)

Additional file 2: The RAD-seq alignments between twenty samples and sample “F2A”. (DOCX 16 kb)

Additional file 3: Agarose gel separation of PCR amplification products with female-specific and control primers in 24 females and 24 males from a full-sib family cultured in a pond of Raoping, China. Female-specific band (320 bp): PCR products amplified with SPFS primers; Control band (282 bp): PCR products amplified with SPC primers; M: marker; A: the results for 12 females and 12 males. B: the results for another 12 females and 12 males. (DOCX 680 kb)

## Abbreviations

AFLP: Amplified fragment length polymorphism
NGS: Next-generation sequencing
RAD-seq: Restriction-site associated DNA sequencing
RAPD: Random amplified polymorphic DNA
SNPs: Single nucleotide polymorphisms
SSR: Simple sequence repeat
