# Supplementary material for: Female-specific SNP markers provide insights into a WZ/ZZ sex determination system for mud crabs Scylla paramamosain, S. tranquebarica and S. serrata with a rapid method for genetic sex identification
Source: BMC Genomics. 2018 Dec 29;19:981. doi: 10.1186/s12864-018-5380-8 (PMC6311006; doi:10.1186/s12864-018-5380-8)
Supplement: Supplementary file 1 — The cluster analysis and RAD assembly of sample “F2A”. (DOCX 14 kb) [file 12864_2018_5380_MOESM1_ESM.docx]

**Additional file 1. The cluster analysis and RAD assembly of sample “F2A”.**

| Sample name | Cluster Tag number | Clean Tag number | Total contig base (bp) | Total contig number | Average contig length (bp) | N50 length (bp) | GC (%) |
| --- | --- | --- | --- | --- | --- | --- | --- |
| F2A | 869,782 | 272,347 | 69,799,531 | 242,137 | 288.3 | 300 | 40.50 |
