# Supplementary material for: Female-specific SNP markers provide insights into a WZ/ZZ sex determination system for mud crabs Scylla paramamosain, S. tranquebarica and S. serrata with a rapid method for genetic sex identification
Source: BMC Genomics. 2018 Dec 29;19:981. doi: 10.1186/s12864-018-5380-8 (PMC6311006; doi:10.1186/s12864-018-5380-8)
Supplement: Supplementary file 2 — The RAD-seq alignments between twenty samples and sample “F2A”. (DOCX 16 kb) [file 12864_2018_5380_MOESM2_ESM.docx]

**Additional file 2. The RAD-seq alignments between twenty samples and sample “F2A”.**

| Sample name | Mapped reads | Total reads | Mapping rate (%) |
| --- | --- | --- | --- |
| F1A | 12921881 | 21847092 | 59.15% |
| F2A | 21260062 | 26462942 | 80.34% |
| F9A | 12539535 | 16501732 | 75.99% |
| F1C | 14113329 | 19460818 | 72.52% |
| F2C | 4560469 | 6843196 | 66.64% |
| F3C | 19303451 | 26567472 | 72.66% |
| F5C | 20375379 | 28275808 | 72.06% |
| F6C | 2650115 | 3886802 | 68.18% |
| F8C | 11970060 | 16356092 | 73.18% |
| F9C | 4737123 | 6687572 | 70.83% |
| M1A | 8792599 | 11718538 | 75.03% |
| M3A | 2785822 | 3924978 | 70.98% |
| M5A | 5534215 | 7514458 | 73.65% |
| M6A | 4923043 | 9898912 | 49.73% |
| M8A | 7830501 | 10442804 | 74.98% |
| M2C | 6267121 | 8790688 | 71.29% |
| M3C | 20268328 | 27115768 | 74.75% |
| M4C | 11850696 | 16257120 | 72.90% |
| M6C | 3479496 | 4944440 | 70.37% |
| M7C | 7656421 | 10727510 | 71.37% |
