# Supplementary material for: Female-specific SNP markers provide insights into a WZ/ZZ sex determination system for mud crabs Scylla paramamosain, S. tranquebarica and S. serrata with a rapid method for genetic sex identification
Source: BMC Genomics. 2018 Dec 29;19:981. doi: 10.1186/s12864-018-5380-8 (PMC6311006; doi:10.1186/s12864-018-5380-8)
Supplement: Supplementary file 3 — Agarose gel separation of PCR amplification products with female-specific and control primers in 24 females and 24 males from a full-sib family cultured in a pond of Raoping, China. Female-specific band (320 bp): PCR products amplified with SPFS primers; Control band (282 bp): PCR products amplified with SPC primers; M: marker; A: the results for 12 females and 12 males. B: the results for another 12 females and 12 males. (DOCX 680 kb) [file 12864_2018_5380_MOESM3_ESM.docx]

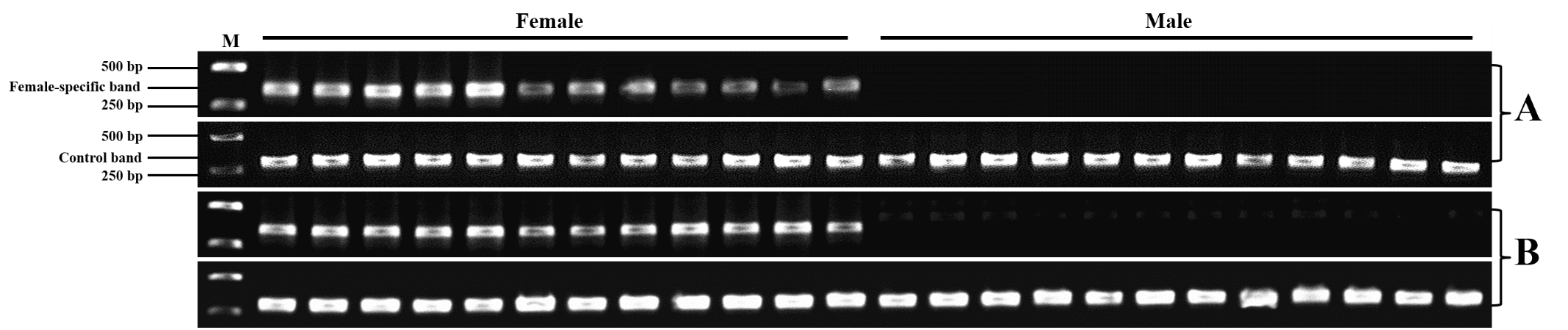


**Additional file 3. Agarose gel separation of PCR amplification products with female-specific and control primers in 24 females and 24 males from a full-sib family cultured in a pond of Raoping, China.** Female-specific band (320 bp): PCR products amplified with SPFS primers; Control band (282 bp): PCR products amplified with SPC primers; M: marker; A: the results for 12 females and 12 males. B: the results for another 12 females and 12 males.
